# Medicalisation, pharmaceuticalisation, or both? Exploring the medical management of sleeplessness as insomnia

**DOI:** 10.1111/1467-9566.12820

**Published:** 2018-09-21

**Authors:** Catherine Coveney, Simon J. Williams, Jonathan Gabe

**Affiliations:** ^1^ School of Applied Social Sciences De Montfort University Leicester UK; ^2^ Department of Sociology University of Warwick Coventry UK; ^3^ Criminology & Sociology School of Law Royal Holloway Egham UK

**Keywords:** medicalisation, sleep, interviewing (qualitative), medical knowledge, drugs/medication, primary care

## Abstract

In this paper we examine the medical management of sleeplessness as ‘insomnia’, through the eyes of general practitioners (GPs) and sleep experts in Britain. Three key themes were evident in the data. These related to (i) institutional issues around advocacy and training in sleep medicine (ii) conceptual issues in the diagnosis of insomnia (iii) and how these played out in terms of treatment issues. As a result, the bulk of medical management occurred at the primary rather than secondary care level. These issues are then reflected on in terms of the light they shed on relations between the medicalisation and the pharmaceuticalisation of sleeplessness as insomnia. Sleeplessness, we suggest, is only partially and problematically medicalised as insomnia to date at the conceptual, institutional and interactional levels owing to the foregoing factors. Much of this moreover, on closer inspection, is arguably better captured through recourse to pharmaceuticalisation, including countervailing moves and downward regulatory pressures which suggest a possible degree of depharmaceuticalisation in future, at least as far prescription hypnotics are concerned. Pharmaceuticalisation therefore, we conclude, has distinct analytical value in directing our attention, in this particular case, to important dynamics occurring within if not beyond the medicalisation of sleeplessness as insomnia.

## Introduction

In this paper we examine the medical management of sleeplessness as ‘insomnia’ in Britain through the eyes of general practitioners (GPs) and sleep experts. The latter group in particular has been given little sociological attention to date. Our paper is located at the nexus of long‐standing debates within medical sociology on the medicalisation of society and other recent scholarship on sleep matters within the social sciences and humanities more generally. We briefly consider some of this recent sociological work with particular reference to medicalisation‐pharmaceuticalisation debates, before turning to an analysis of the key themes in our data and discussion of what further light this sheds on the medicalisation and pharmaceuticalisation of sleeplessness as ‘insomnia’.

### Insomnia

Whilst sleeplessness has been with us through the ages, ‘insomnia’, *qua* medical term of reference, is a more modern matter: a term which became increasingly common in medical parlance from the late nineteenth century onwards (Ekirch 2015). Although meanings and definitions of ‘insomnia’ vary within medical and popular culture, both past and present, this is commonly taken to mean difficulty falling asleep or staying asleep, early morning awakening, sleep dissatisfaction and daytime consequences such as tiredness. All of these may be short‐term or long‐term in nature (NHS Choices 2015). A significant development here comes in the shape of the most recent iteration of the Diagnostic and Statistical Manual, the DSM‐5 (American Psychiatric Association 2013) which explicitly refers to ‘insomnia disorder’ thereby marking a move away from previous classifications where it was demarcated as being either ‘primary’ (or independent from) or ‘secondary’ (as symptom) to another disorder.

Estimated prevalence of insomnia in the UK varies from 10‐48%, depending on which population is being studied and the definitions of insomnia adopted in each study (Morphy *et al*. 2007). It has recently been reported that up to one‐third of Britons are suffering from insomnia at any one time, and that at least one in ten can be characterised as a chronic insomnia sufferer (MHF 2011).

Reports indicate that around a tenth of people in the UK take sleeping tablets three or more times a week and that the likelihood of taking them increases with age (Understanding Society, 2011). In 2016, approximately 9 million prescriptions for hypnotics were dispensed in the community in England at a cost of over £52 Million (Health and Social Care Information Centre, 2017). Although hypnotics are still being prescribed in great numbers in England, prescription rates have been moderately falling over recent years, after a period of stability where they remained at around the 10 million per annum rate from 2004 to 2012.

However, prescription rates and figures can only give a partial picture of what is happening and should be approached with caution. For example, prescriptions dispensed in hospitals or secondary care clinics are not included in the above figures, which also only consider England as opposed to the whole of the UK. In addition, we cannot tell from these data whether the prescription was a repeat or for a new user, so the number of prescriptions dispensed in the community does not equate to the number of people using hypnotics. A further issue to consider is that people may have been diagnosed with insomnia and prescribed sleeping pills but decided not to take them – several sociological studies across different populations have shown that hypnotics are regarded as a last resort and are frequently rejected by those with sleep problems (Cheung *et al*. 2017, Gabe *et al*. 2016, Hislop and Arber 2003, Venn and Arber 2012).

Recent strands of work within the sociology of sleep have sought to explore how ‘insomnia’ or ‘poor sleep’ is managed in every day/night life, far away from the doctor's surgery or the sleep clinic (Venn and Arber 2012, Venn *et al*. 2013). Across these studies, poor sleep was often regarded as a normal or inevitable consequence of daily life and people tended to express negative beliefs about sleeping pills and their effects, thereby providing grounds to ‘resist’ medicalisation and pharmaceuticalisation of their sleep. The moral dimensions of these matters have also recently been explored by Gabe *et al*. (2016), who identify a range of ‘moral repertoires’ which people draw upon when discussing their use and non‐use of sleep medicines.

Other studies have attempted to understand doctors’ motives and explanations for prescribing sleeping tablets (Davy *et al*. 2015, Gabe and Lipshitz‐Phillips 1984, Moloney 2017, Sirdifield *et al*. 2013). These works draw attention to high levels of ambivalence around the prescription of sleeping pills. Tensions exist in doctors wanting to help patients, avoid confrontation and demonstrate empathy through the prescription of hypnotics, while acknowledging the potential for reliance on sleep medicines ‐ which are typically not regarded as a first line therapy for insomnia ‐ and accepting responsibility for minimising the use of these drugs. For Moloney (2017) what this amounts to is a case of ‘reluctant medicalisation’ for physicians and patients alike.

To further understand the medical management of sleeplessness as insomnia, the next section turns to sociological questions of medicalisation, pharmaceuticalisation, and relations between them as discussed and debated so far in the sociological literature, with sleeplessness or ‘insomnia’ in mind.

### Medicalisation and pharmaceuticalisation

In terms of medicalisation, we draw on Conrad's (1992, 2007) work on medicalisation, which denotes the ‘making medical’ of some hitherto ‘non‐medical’ matter. This, Conrad stresses, is not an all or nothing process but may occur at different *levels* – *conceptually* in the way a problem comes to be understood in medical terms, *institutionally* in the way that it comes to be managed, *interactionally* through the doctor‐ patient relationship – and to varying *degrees*, including the potential for demedicalisation over time. Medicalisation, then, is a *bidirectional process*, in principle if not always in practice. Medicalised categories in this respect, as Conrad's (2007) own case studies suggest, may ‘expand’ or ‘contract’ over time with definitional matters still at the centre of this process.

Three key points follow. First, the importance following Conrad (2007: 4‐8) of *definitional matters* when considering cases of medicalisation. Second, that a more *complete*, rather than partial, case of medicalisation would involve medicalisation at each of the three levels identified above, with little or no resistance or refusal on the part of those involved in these processes. Thirdly, and equally importantly, recourse to the ‘medicalisation of insomnia’ – which as we shall see below is not uncommon in the current sociological literature, even by Conrad himself (Conrad *et al*. 2010) – is something of a tautology, given that it is really the medicalisation of sleeplessness (‘difficulty’ sleeping, ‘poor sleep’ or some such colloquialism) as insomnia which is at stake here.

The second related yet distinct concept we draw on this paper is *pharmaceuticalisation* which, in similar fashion, denotes the ‘transformation of human conditions and capabilities into the opportunities for pharmaceutical intervention’ (Williams *et al*. 2011: 711). Pharmaceuticalisation, as such, directs our attention more specifically to the role of pharmaceuticals *within and beyond* these medicalising processes (Abraham 2010).

Our analysis connects to wider ongoing debates regarding the overlaps and intersections between medicalisation and pharmaceuticalisation, including whether pharmaceuticalisation is a useful concept in of itself or can be subsumed within medicalisation. A full rehearsal of these debates (Busfield 2017a,b, Williams *et al*. 2017), including the not insignificant matter of what counts as medicalisation in the first place, if not medicalisation*s* in the plural (Correia 2017) is beyond the scope of this paper. Suffice it to say, for present purposes, that the position we are taking here follows others who argue that each of these concepts may vary independently of one another in complex, dynamic ways (see Abraham 2010, Bell and Figert 2012, Williams *et al*. 2011, 2017) shaped in part by countervailing pressures from a variety of interdependent actors seeking to pursue their own interests (Light 2010).

Whilst medicalisation then, may occur in the absence of pharmaceuticalisation, pharmaceuticalisation may also arguably occur in the absence of, or far outstrip, the medicalisation of a problem at any particular point in time, as the case of pharmaceutical enhancement amongst the otherwise healthy suggests (Coveney *et al*. 2011). Alternatively, the medicalisation of a problem may continue in the midst of a slow or rapid process of *de‐*pharmaceuticalisation (Gabe *et al*. 2015, Williams *et al*. 2017), including the potential for doctors or other experts to want something akin to ‘more’ or ‘better’ medicalisation but less pharmaceuticalisation. The value of pharmaceuticalisation, then, is not solely to do with those instances which extend *beyond* medicalisation but also those occurring *within* medicalisation, including the depharmaceuticalisation of a said problem over time, even if it remains medicalised to some degree.

Here we arrive at the following research questions which we seek to answer in the remainder of this paper, regarding sleeplessness in general and sleeplessness as insomnia in particular:

1. What do GPs and sleep experts have to say about the medical management of sleeplessness in Britain today?
2. What does this tell us about the medicalisation of sleeplessness as insomnia?
3. What further analytical light, if any, does pharmaceuticalisation shed on these matters?
4. Are there, reciprocally, any signs of the demedicalisation and/or depharmaceuticalisation of sleeplessness as insomnia and, if so, what do they amount to?

## Methods

Data were collected as part of a wider study looking at Pharmaceuticalisation of Sleep and Wakefulness in the UK since 2000, funded by the UK Economic and Social Research Council. Following ethics approval from the National Health Service (NHS) in England we interviewed 7 sleep (medicine) experts (5 sleep scientists and clinicians, 2 psycho‐pharmacologists) and 7 General Practitioners, between April 2013 and May 2014.

Sleep experts were invited to participate in the research on the basis of their reputation in this field, their interest in sleep problems and experience of sleep management. Our aim was to interview individuals with expert knowledge who could act as key informants for our research, not to obtain a large or representative sample. The sampling frame that was developed and used had two components. First, we conducted a mapping exercise where we identified all UK sleep clinics and stratified these according to the type of patient they saw (e.g. respiratory sleep disorders/non‐respiratory sleep disorders). We also mapped membership and leadership roles taken in professional societies and on the board of key academic journals. Second, we conducted a literature search of sleep medicine journals and media sources to identify organisations and individuals who dominate the public domain in relation to sleep medicine in the UK. The cross‐referencing of these data sources allowed us to identify a list of 16 high profile individuals with expertise across different domains of sleep medicine to approach to participate in the research. We asked for an interview with them and for access to their clinic patients if they were based in a sleep clinic. This resulted in 7 interviews, with two clinics allowing us access to their patients. Consequently, for our analysis, following Mitchell (1983: 207), it can be said that ‘the validity of the extrapolation’ is dependent on the ‘cogency of [our] theoretical reasoning’ and the extent to which the data support this reasoning.

We use the term ‘sleep expert’ in the paper as an intentionally broad catch–all label in order to protect the anonymity of our research participants. Five other ‘experts’ from patient and advocacy groups and other specialist academics were interviewed as a part of the study but are not included in this paper as their expertise and experience were not directly relevant to the medical recognition and clinical management of sleeplessness.

GPs were recruited via the Primary Care Research Network (PCRN).^1^ The call for participants was distributed to all primary care practices within two Primary Care Trusts. Those who were interested in participating in the research were encouraged to contact the PCRN liaison, who then put them in touch with the research team. Ten GPs initially came forward, 7 of whom were interviewed owing to 3 subsequent non‐responders. We stopped collecting data once our leads were exhausted. Participant demographics are described in Table 1.

**Table 1:** Participant demographics

General practitioners	M/F	Size of practice	Location of practice	Length of time as GP
GP1 ‐7	4 M 3F	From 3,900 patients to 13,000	2 Rural 5 Urban	0‐5 years (1) 11 – 15 years (4) 25 + years (2)

Sleep medicine experts	M/F	Broad area of expertise
EI1	F	Sleep clinician, biomedical
EI 2	M	Sleep scientist, psychological medicine
EI 3	M	Sleep clinician, biomedical
EI 4	M	Sleep clinician, biomedical
EI 7	M	Psycho‐pharmacologist
EI 8	F	Psycho‐pharmacologist
EI 10	M	Sleep scientist, psychological medicine

Interviews were carried out by one or other of the authors and took place at the interviewee's place of work or at the university where the project was based. Topics covered ranged from their views on sleep management, including sleep medicines, the treatment of particular conditions, the future of drug developments and policy and practice regarding sleep matters. Interviews lasted for around an hour on average and were audio recorded and transcribed. Analysis of the transcripts was facilitated using the qualitative data analysis software package NVivo 10. We took an inductive approach to data analysis which involved reading and re‐reading the transcripts, grouping data extracts together based on the main themes and generating a coding frame to identify major topics and issues and grouping these together into themes. Codes and themes relating to major issues were discussed between the authors for purposes of reliability and validity. On this basis we developed an interpretative analysis of the meaning of insomnia and its management and drew out the implications for the research questions outlined above, with particular reference to medicalisation and pharmaceuticalisation.

## ‘Insomnia’ Today: problems and prospects

Through our analysis of the sleep expert and GP data, we generated three key themes concerning the problems and prospects of sleeplessness as ‘insomnia’. These related to: (i) institutional issues around advocacy and training in sleep medicine; (ii) conceptual issues in the diagnosis of insomnia; (iii) and interactional and practical issues in insomnia management, both in terms of pharmaceuticals and alternatives. It is to these key themes that we now turn.

### Institutional issues

First, we discuss institutional issues relating to the status of sleep medicine in the UK, including organisation, training and sleep literacy.

#### Advocacy: Who ‘owns’ insomnia?

An important theme concerned what might collectively be termed problems of ‘advocacy’ and ‘ownership’ regarding sleep in general and insomnia in particular. In part, this pertained to commonly voiced concerns regarding the organisation and status of sleep medicine compared to other areas of medicine, particularly in the UK.

In the data extract below, one of the sleep experts we interviewed talks about the lack of provision of sleep medicine services in the UK, contrasting this to the US where sleep medicine is more established as a speciality. The latter is partly to do with funding and the organisation of healthcare, and partly to do with the priority or recognition given to sleep problems:

Respiratory sleep medicine is very well established and they've done brilliantly, we have a lot to learn from them. It's the non‐respiratory which is very limited […] I think we've got half a dozen neurologists and people like me interested, a couple have come from psychiatry […] There are very few and a very limited resource, even, in the centres that are […] we can't meet the patient demand […] I think Americans are miles ahead […] they are far more well‐established in non‐respiratory sleep disorders than over here […] I mean, if [the commissioners] had to choose, would they really consider sleep disorders or would they consider heart or cancer? I think if they have to make a decision on supporting a sleep medicine centre or something, they might have a difficult choice. (EI 1)

However, the organisation of sleep medicine in the US does not equate to patients presenting with insomnia automatically being referred to a sleep clinic or specialist either. Recent research in the US has shown that access to specialist clinic services for insomnia can, to a large extent, depend upon ‘the richness of resources in one's social network and surrounding environment rather than the clinical severity of the disease alone’ (Cheung *et al*. 2017: 659). In part, however, lack of access to a specialised centre is related to insomnia as a particular problem, compared to say obstructive sleep apnoea (OSA) or narcolepsy, which are better catered for in the UK if not ‘owned’ through respiratory medicine or neurology. Consider, for example, the following quote from one sleep expert who spoke at length about these matters:

There's no medical specialty that feels an ownership affinity with insomnia. […] And that's problematic for people with insomnia because it means when they present to the GP (there's no) pathway […] You see, when NIC^2^ guidelines for CPAP^3^ were published everybody knew that if we published this guideline then we're empowering respiratory medicine to do what they are doing. But, if you publish a guideline that says this is how you should treat insomnia, who are you talking to? Are you talking to the IAPT^4^ agencies; are you talking to clinical psychology; are you talking to psychiatry? (EI 10)

That is not to say that other sleep disorders have been medicalised and that these sleep disorders are diagnosed, managed and treated unproblematically, which is far from the case (see, for example, Zarhin's (2015) work on Sleep Apnoea). However, the management of these disorders contrasts with insomnia where clear treatment pathways to secondary care clinics do not exist, leaving chronic insomnia patients with nowhere to go beyond general practice. GPs, too, discussed how without the secondary and tertiary care pathways that exist for other sleep disorders, insomnia patients become a somewhat ‘neglected group’:

I think [sleep] is an area which is probably a little bit neglected for sort of referring onwards. Most things, once you've tried your little band of things, there's something else you can get, and as I said I've come across these two patients who've seen the psychiatrist. But again, they're not really a sleep specialist. You kind of almost need a sleep department where there's [someone looking at all of the different aspects] … maybe a psychiatrist, psychologist, maybe chronic pain because that might get in there as well, something along those lines but there isn't […] I know that we don't, some doctors can arrange the sleep studies but it's not like there's a sleep disorder unit [around here] and again they're more interested in sleep apnoea and those type of things, that they can physiologically can pick up. This group [insomnia patients] are a neglected group. (GP 3)

We can see in the data extract above how the medicalisation of insomnia at the organisational level is both partial and problematic. Although patients are presenting with insomnia to primary care, clear treatment pathways are not in place for these patients to progress through the medical system to access more specialised care. Sleep medicine can be considered a niche if not ‘Cinderella’ area in the UK still, with provision of and access to sleep clinics being patchy and localised, led by a small number of interested researchers and clinicians. Without strong advocates from within the medical profession or organised care pathways beyond general practice, insomnia patients then can fall between the cracks and occupy a somewhat *liminal space*, being left with only their GP to turn to.

#### Education: a case of poor sleep ‘literacy’?

The above issues in turn merged with sleep experts’ concerns regarding a lack of basic education and awareness about sleep matters and sleep medicine in general, both inside and outside medicine. GPs were frequently mentioned here, through no fault of their own, as in need of better education and training in basic principles of sleep and sleep medicine. It was argued that, while doctors would become well versed in pharmacology, medical training often did not focus on non‐pharmacological treatments such as cognitive behavioural therapy for insomnia (CBTi) and how this might be beneficial in insomnia management.

The medical profession knows very little about sleep and sleep problems. It's just a neglected topic because nobody takes responsibility for it […]. I think the whole medical training doesn't focus on non‐pharmacological methods of treatment. Going back [to] the brilliant developments in the ‘30s and ‘40s it was important to make a diagnosis because we were developing specific treatments. We've done all that and we've got to pretty well an impasse with that in various areas of medicine. The idea that somehow we need to look at less focused methods of treatment, although CBT(i) is focused of course and GPs, especially the older ones, have not been exposed to this sort of thing […], that's the impression I get speaking to the graduates who come through here. If I lecture on insomnia and I say, “Who's had lectures and seminars on it?” Not a hand goes up. […]. You don't come across insomnia experts except in just a few very privileged places. (EI 7)

As one sleep expert put it, this could leave GPs knowing more about the pharmacology of the drugs they are prescribing than the condition they are prescribing for:

We have a situation now where we have doctors who […] know the pharmacological properties of sleeping drugs, they are very familiar with the half‐life, with the clearance rate, they know the molecule, they know its toxicity level, they know its therapeutic dose. They know lots about these drugs; they're doctors, they're meant to know a lot about them. These same doctors would struggle to explain how people come to fall asleep under normal circumstances. Now, that's a bit bizarre … . But, we tolerate this in general practice where we've got doctors who know lots about sleeping tablets, prescribing for a condition that they really would struggle to articulate on. That's not saying that this is somehow a fault of theirs. It's cultural. (EI 10)

This lack of knowledge of sleep medicine in turn led to concerns being raised regarding the recognition given to insomnia as an illness in its own right in primary care settings:

I think because of the lack of a speciality in sleep medicine, and lack of training in sleep, people don't quite appreciate it as an illness. I think symptoms are recognised and almost certainly plague GPs but giving a sleep related symptom a diagnosis, I think people tend to shy away from that. They just accept it as a symptom, possibly lifestyle related when it isn't … I think certainly GPs are unconfident about diagnosing and what to do about sleep medicine in general, actually, or the lack of clinics and the lack of education. (EI 4)

We asked GPs if they thought that sleep problems were widely recognised as medical problems by the medical community in the UK. Their responses indicated that sleep is on the agenda in primary care, but predominantly in relation to the ways in which sleep problems are associated with other illnesses, particularly mental health issues. Training in how to manage sleep problems is inevitably encountered when working in the GP surgery and dealing with the complexity of patients medical complaints:

I Do you think sleep problems are widely recognised by the medical community as medical problems in the UK?

GP3 Yes I think people, you know, it's sort of seen, and certainly we've been, I can remember being taught as a medical student and as a trainee doctor ways to manage it. I sometimes think the ways that we have to manage it maybe aren't very successful. We help up to a certain level but I'm sort of thinking about my two intractable insomniacs and it's very difficult.

I You mentioned your training. It gets said that medical students don't get really trained in sleep …

GP3 I think people would have mentioned, maybe not as a medical student, maybe more as a trainee because, particularly in GP training you're starting to see the patients that you realise that you're going to be actually seeing […] you start looking and realising that these difficult things are going to be on your doorstep and you're going to have to come up with some solutions to know what to do. I'm trying to think as a student, I'm sure it must have come up somewhere. I can't think. (GP 3)

As mentioned above, the lack of formal organised care pathways for insomnia patients and scarcity of specialised sleep clinics in the UK consequently results in the responsibility for the medical management of insomnia falling more by default than design to GPs in the main. Such GPs, when they encounter complaints of sleeplessness or sleep disturbance, may or may not go on to *diagnose* insomnia. As our data show, there are concerns that inattention to sleep matters in medical education may be leading to a lack of understanding and lack of recognition of insomnia (and sleep problems more generally) and limiting therapeutic options predominantly to the pharmacological rather than newer but less well known psychological therapies. Thus, our data further problematise the medicalisation of insomnia both at the conceptual and interactional levels.

### Diagnosis: symptom or disorder?

A second key theme, evident in both our sleep expert and GP interviews alike, concerned the very nature and status of ‘insomnia’ itself as symptom or disorder. As E18 asserts below, most, but not all, of those sleep experts interviewed recognised that insomnia can exist as a *disorder in its own right*, sometimes exhibiting comorbidity with other disorders and sometimes being implicated as a part of them.

[Insomnia] can sometimes be a symptom of depression and then often depressed patients’ first presentation to primary care is because of their sleep problems. But, people who are depressed can also have insomnia and sometimes that goes on, even when their mood gets better and so there's no reason not to treat that, really. So I think this focus on it's one thing or another thing; it's a disorder. Insomnia's a disorder. You need to think about treating it because it's going to prolong … if people get better from depression but still have insomnia, they're at much more risk of recurrence so there's no reason not to treat it.

Interviewer Do you think there needs to be more recognition of insomnia as a disorder in its own right?

Yes, I do. (EI 8)

Some sleep experts, such as EI 2, described the recent shift in DSM‐5 to ‘insomnia disorder’ as welcome:

I think the DSM‐V criteria represent or reflect a better literature now that, in fact, sleep disturbance is a more primary issue rather than a secondary issue and probably the fairer thing to say is – when one doesn't know if it's primary or secondary – regard them as disorders in their own right that merit treatment, merit intervention in their own right but I'm not sure that the average GP would see it that way. (EI 2)

Others spoke of the complexities and difficulties ‘insomnia’ presented in clinical practice, particularly in the presence of conditions such as depression and saw value in keeping a primary/secondary distinction:

I do find it useful, the old classification […] that division for me as a clinician is helpful to explain to patients that, you know, “You had some trigger but the trigger is then resolved.”[…] Having said that […] I can see where some professionals may find it useful not to have this division but in a service like ours, it is useful to tease out those who don't have major psychiatric disorders but have significant insomnia and find that extremely frustrating, who psychiatrists won't see and, therefore, need some help from somewhere else and some explanation of their condition. (EI 1)

Similarly, for many of the GPs we spoke to, insomnia was typically divided into primary insomnia, where the sleep problem was viewed as the main complaint and no other underlying cause could be identified, and secondary insomnia where the sleep problem was considered to be a symptom of another illness or disorder or resulting from lifestyle/behaviour stressors.

Most of them are mainly associated with depression. Rarely (do) I see a sleep disorder per se. I would say at least, if you want to say sleep, like once a month or something. But most of them are associated with some other disorder […] Depression, yeah, or anxiety. (GP 7)

Instances of primary insomnia as this suggests were considered to be rare amongst the GPs we spoke to, whereas secondary insomnia was common and often linked to mental health problems such as depression or anxiety. A range of lifestyle and behavioural factors were also often mentioned in relation to secondary insomnia. GPs, for example, spoke about issues such as bereavement, shift work, travel, stress, substance abuse, caffeine consumption, pain, noise, redundancy or money worries, family conflicts, and caring for others, as common factors affecting their patients’ sleep.

If I classed it as primary/secondary […] primary where there would be no associated or underlying conditions; that it wasn't due to a physical nature or perhaps a sort of psychological issue. So, from a secondary point of view, I would say I saw a lot of sleep problems. I can't quote you exact figures […] if I think back that I'd probably say at least once a day that one of the patients I saw would have had a sleep related issue, from a secondary point of view, as a result of something that they were having to deal with […] If I look back to when I moved down to [city] and I was there for two years, and I can honestly say there was only one person that I felt fulfilled the criteria for primary [insomnia]. (GP 2)

Insomnia, then, seems to occupy a liminal space in medicine where it can be simultaneously regarded as both symptom and disorder at any one time. This ‘fuzziness’, for want of a better word, in conceptualisations of insomnia and the ways in which it is related to other medical issues or disorders impacts on the way in which sleeplessness comes to be medicalised. Whether occurring independently from, co‐morbid to or as a part of (an)other disorder(s), sleep experts tended to view insomnia as an important health concern that should be recognised and treated in its own right. In comparison, at the primary care level, many GPs continue to view insomnia, in the main, as occurring as a secondary symptom of other disorders, particularly mental illness, as opposed to giving insomnia recognition as a discrete disorder in its own right that is equally deserving of medical attention and treatment. The GPs in our study demonstrated reluctance to diagnose primary insomnia (using their terminology). This was reflected in the low number of patients we were able to identify who had been diagnosed with insomnia in the GP practices we researched. Some of the GP practices we gained access to did not have any patients with an insomnia diagnosis at all, most had only a handful, despite sleep complaints being one of the most prevalent complaints GPs said they were faced with on a daily basis. Few patients it seems, then, will be given a diagnosis of (primary) insomnia through interaction with their GP. This in turn contributes to the partial medicalisation of sleeplessness as insomnia conceptually speaking and has implications for the ways in which it comes to be managed.

### Treatment: keep on taking the tablets?

Here we consider the final key theme in our data concerning the role of pharmaceuticals *vis‐à‐vis* other non‐pharmaceuticals interventions in the management of sleeplessness within medicine. This relates to how sleeplessness is medicalised and/or pharmaceuticalised through treatment practices.

Current guidance for insomnia management in the UK is to identify and manage the underlying cause of insomnia (if one can be identified); to advise good sleep hygiene and to only prescribe a hypnotic if daytime functioning ‘is significantly impaired’. This should be for a period of no more than 2 weeks. GPs are instructed to ‘review after 2 weeks and consider referral for cognitive behavioural therapy if the symptoms persist for longer than 4 weeks’ (NHS Choices 2015). Pharmacological therapy is generally not recommended for the long‐term management of insomnia. Instead good sleep hygiene, exercise and referral to psychological therapies are listed as possible treatment routes (NHS Choices 2015). The guidance does note that the patient could be referred to a specialised sleep clinic for insomnia management if the symptoms persist, but states that ‘access to sleep clinics depends on locality’ (NHS Choices 2015).

#### Pharmacological management of sleeplessness

Some of the sleep experts we spoke to were of the opinion that lack of training in sleep medicine and alternatives to pharmaceuticals may constrain GPs to act within their ‘clinical comfort zones’, as one expert put it, perhaps prescribing hypnotics out of familiarity, habit or even ‘laziness’:

I don't believe in the long‐term prescription of anything except for something that is going to literally save your physical life.

Interviewer Well, what do you think the factors are that we have this kind of pattern [of high levels of hypnotics being prescribed]?

That could be … Lack of availability of skilled resources that can treat insomnia in the beginning, laziness by primary care providers who would just rather write a prescription. (EI 3)

GPs, on the other hand, described a reluctance to prescribe hypnotics in moralised terms, presenting themselves as ‘low prescribers’ and hypnotics as a last resort and a short‐term measure. They also expressed concern regarding cases of sleeping pills first prescribed in secondary care that they had to manage:

A lot of the Z drugs come out of secondary care and they work very well but they're only licensed for short‐ term use. Secondary care knows this, but they have a tendency to tell the patient, “go and see your GP for more”. Unfortunately we then tell them, “actually, it's only for short‐term use; we have a policy that we don't prescribe”. (GP 6)

They often spoke about other medicines (with sedating effects) that they prescribed *instead of* hypnotics, such as sedative antihistamines, painkillers, antipsychotics and antidepressants. This prescribing was typically viewed as treating the underlying *cause* of the problem, not just the *symptom* of insomnia. However, it was also common for GPs to say they would prescribe a short course of hypnotics *alongside* another medication (e.g. antidepressants) while waiting for the other medication to take effect.

It would be very short term, always. So, like an acute stressful situation, loss of a relative, or things like that. I might use it short term in people where they are depressed, and you are trying a sedative anti‐depressant and they're not sleeping well. Initially, you might just get them through that hurdle while medication takes effect […] I'm a very low prescriber of sleepers I am, very low. I obviously sign the repeats every day, I can't think, it must be maybe one a month or something I'm signing a repeat prescription for sleeping [tablets]. Mostly it is acute, just one off. So, I don't understand [why prescription rates have remained stable] unless it's a certain generation of GPs, maybe, older generation are more lax with it. I think you'll find the young ones coming through are more in tune with my line of thinking. So, hopefully, it will be a trend down at some point. (GP 1)

GPs also discussed advising patients on matters of sleep hygiene and/or advising the purchase of an over‐the‐counter (OTC) sleeping aid before prescribing hypnotics. This could therefore, according to the sleep expert quoted below, amount to a doubling of the ‘real pharmacology of night‐time sleep’, once OTCs were also counted:

I think, last time I looked it was about 10‐11 million hypnotics a year. Now, the numbers depend on how you define hypnotics, whether you pop in melatonin or you take out melatonin or whether you include over‐the‐counter medications that people access which we believe to be at a level that matches prescribed drugs, or whatever is prescribed, you can double it for the real pharmacology of night‐time sleep. And, many of those things that are consumed aren't herbal nonsense, they are actually effective drowsiness inducing drugs of the old antihistamine variety. (EI 10)

Whilst sleep experts and GPs alike all recognised a role for sleeping pills in the medical management of insomnia, short‐term or otherwise, they also spoke at length about the problems of managing insomnia, given the aforementioned themes and issues, particularly in primary care.

The *temporal* dilemmas and uncertainties of managing insomnia through drugs were mentioned in various ways. One such dilemma practitioners are faced with as noted above, is that the hypnotics currently available for prescription are licensed for short‐term use only. This is invariably at odds with the cases of insomnia that persist for longer than the recommended 2‐4 weeks of medication use and the difficulties in assessing how long a sleep problem is likely to persist for:

Most insomnias that fetch up in primary care settings are chronic insomnias […] patients don't have a week's poor sleep and go to their doctor, as a rule. They suffer for a long time. So, by the time they are presenting their symptoms, their symptoms have already been described as having a history that is out with the prescribing guidelines of the tablets. If you say “look, I've had this problem for three months now, I can't stand it anymore”. And the doctor says “oh, I've got this solution, it lasts for three weeks.” Well, realistically, how likely is it that this problem is going to go away in three weeks? […] At one level it's completely unreasonable. There aren't many drugs that we're told “you can have it for three weeks and by the end of that period we presume it will go away”. How would you know? It doesn't make a lot of sense. (EI 10)

GPs were all too aware that the sleeping pills currently available are licensed for short‐term use only. They spoke at length about restrictions and constraints placed on prescribing hypnotics in primary care, and the implications this had on their prescribing practices. This included reference to both local and national measures in place in attempts to reduce the number of hypnotics being prescribed. Some spoke of surgery policies not to prescribe hypnotics for longer than 4 weeks (or 12 weeks for melatonin to the over 50s) and many were actively trying to reduce their prescribing rates. Several different measures to reduce hypnotic prescribing were being implemented in this respect across the GP surgeries we researched. These included individual surveillance and peer comparison processes where each GP's prescribing practices were monitored, compared and reported by the practice in an attempt to highlight individuals who were prescribing more than their colleagues; not initiating new hypnotic prescriptions and inviting patients in for a medication review in an attempt to bring the conversation round to coming off long‐term medication.

Within [city name], we have been monitored by prescribing indicators. [One of which] is to decrease the use of hypnotics in the community. As part of last year's target, our aim was to decrease the number of people or the amount of prescriptions we issued for hypnotics which included Temazepam, Zolpidem, Zopiclone […] Basically, we are not initiating anyone who has sleep disturbance or insomnia on medication because of their addicting properties. And people who are already on these medications are being monitored. These medications are not prescribed on a repeat prescription basis. They are put on as acute. That means patients need to ring us every month to go through their symptoms and we are trying to decrease the number of tablets they take. (GP 4)

These attempts to reduce hypnotic prescribing in primary care could lead to further problems and differences of opinion with secondary care providers:

It's very difficult because … there are patients who have been on a stable dose for years and then suddenly see a new GP who says, ‘I'm not prescribing’ and then you have to be pragmatic and say, you know, ‘is it really worth altering their quality of life when they've been on a relatively low dose?’ … and it does become difficult and then I have to support (the patient) and say well, it's not the right (decision) … or sometimes they're going through a lot of stress and that's not the right time. (EI 1).

Here again then we see the temporal dimensions of these matters at play, notably in terms of the relative merits of short‐term versus long‐term use of prescription hypnotics across the primary‐secondary care interface. Hinted at here too are the moral dimensions and dynamics of these matters. These moral dimensions therefore are not simply confined to patients own ‘moral repertoires’ of use or non‐use of these drugs in everyday/night life (Gabe *et al*. 2016) but can also be found in primary and secondary care providers’ own justifications for prescribing or not prescribing these drugs, whether short or long‐term. The pharmaceuticalisation of sleeplessness as insomnia then is a moral as well as a medical matter for patients and professionals alike, especially when it comes to cases of chronic or long‐term hypnotic prescribing.

#### Alternatives to pharmaceutical treatment

A related issue was the perceived *lack of choice* regarding effective alternative treatments to pharmaceuticals in the management of insomnia in primary care, where resource problems were again mentioned by sleep experts:

People still don't get any – it's about choice amongst available effective alternatives. It's not being anti‐medication by any means, but it really is absurd that people don't have the option, GPs don't have the option when [CBTi] might be preferred and arguably more effective for longer‐ term benefit than sleeping pills. (EI 2)

Whilst GPs also spoke about how they would refer patients for CBT if appropriate, this was usually for mental health problems such as depression rather than specifically for insomnia, as noted earlier. There was moreover a notable lack of awareness about CBTi as such amongst the GPs we spoke to and none were familiar with digital CBTi apps that are now available to prescribe and download via the NHS health apps library.

This is [name of region] IAPT service so you can do some CBT on the computer, so you can access it that way if you wish. I guess there's sleep on that, I don't know. It doesn't mention sleeping on there. Oh yes, sleeping problems. So, on their website they have a self‐help guide for sleeping problems. That's 24 pages long […]. My feeling is most people who are having sleeping issues, it's not purely sleep. It's something else. Secondary to something. So, yeah, it's giving people the information, but they have to go off … I always feel there is some responsibility for them and it's not all down to me. I'm not cash and carry; I'm not McDonald's that you come in and just get the answer. That's the IAPTs^5^ leaflet they'll get from me and there's the website address and they can access what you've just seen on that. So, that's the kind of thing I would be giving out for that. (GP 1)

All in all, then, whilst the *judicious* use of prescription hypnotics was recognised by sleep experts and GPs alike, sleep experts tended to see GPs’ continuing prescription of hypnotics as symptomatic of the problems of insomnia management today and the need for better training and resources, including the development of national clinical guidelines on insomnia management and wider access to CBTi. GPs in contrast typically described themselves as ‘low prescribers’ given new prescribing policies and practices at the national and local levels. When GPs spoke of IAPTs services, this was mainly for conditions such as depression, as the underlying cause of insomnia, rather than for insomnia as such. The temporal and moral dimensions and dynamics of these issues are also important to stress here, as we have seen, with short‐term use of these drugs generally regarded as more legitimate than their long‐term use, particular in today's medical and moral climate of downward pressures on prescription hypnotics.

## Discussion

Let us now return to our research questions. What further light do our data cast on these questions and where does this leave us in relation to the literature we reviewed earlier on these matters?

Clearly as far as the first question goes, the sleep experts and GPs in our study discussed a number of problems with the management of sleeplessness, including what we have termed issues of an ‘institutional’ (advocacy and education), ‘conceptual’ (insomnia as symptom or disorder) and ‘treatment’ (pharmaceutical or non‐pharmaceutical interventions) kind. This in turn, as we have seen, meant that the bulk of insomnia management, more by default than design, occurred at the primary rather than the secondary care level. Our data revealed a lack of ‘ownership’ of insomnia in medical practice, the absence of referral pathways for chronic insomnia and limited treatment options, signalling areas where reformulation of health policy could have significant benefits for patients and practitioners alike.

In terms of the question therefore as to whether or not sleeplessness has been medicalised as insomnia and if so to what extent, the answer it seems, contra Busfield (2017a,b), is that the *medicalisation that has occurred is both partial and problematic to date at the conceptual, institutional and interactional levels*. At the conceptual level we have seen how insomnia is somewhat ambiguous if not liminal, oscillating not just between ‘normality’ and ‘pathology’ as Moloney (2017) suggests, but also *between symptom and disorder*. At the institutional level, we have seen how poorly served insomnia is given, as one of our sleep experts memorably put it, no one ‘owns’ insomnia as a clinical specialism. These problems in turn, to a degree, are reflected and reproduced at the interactional level, given the aforementioned conceptual ambiguity of insomnia and the limited resources to manage it effectively, particularly when it is of a chronic rather than short‐term nature. Regulatory factors are also important to mention here (Cloatre and Pickersgill 2014), as they too have a critical bearing on how insomnia is managed and understood. At the *macro* level the attempt to limit and reduce resort to prescription hypnotics is manifested mainly through NICE's recommendations for behavioural and psychological therapies as first‐line interventions. This in turn, at the institutional (*meso*) level, translates into the prospective reduction of prescription hypnotics, the adoption of restrictive prescribing policies, surveillance of GPs’ prescribing practices and top‐down monitoring, as some of our GPs suggested.

These findings of partial and problematic medicalisation are unsurprising perhaps, given previous sociological research which suggests a degree of resistance to the medicalisation of poor sleep amongst prospective patient groups (Venn and Arber 2012, Venn *et al*. 2013). Even in what may at first glance appear a more clear‐cut case of medicalisation in the form of obstructive sleep apnoea (OSA), there is still a considerable degree of doubt over diagnosis and contestation regarding its medicalisation at the micro level, as recent research suggests (Zarhin 2015). Maybe indeed, in a more speculative vein, there is something particular, if not peculiar, about sleep, beyond the factors already discussed, which makes it especially resistant to medicalisation: its intensely moral nature perhaps as a supposedly ‘private’ matter involving conscious responsibility for an unconscious state.

Two further tentative points follow in response to this first question, both of which require further investigation. First it is tempting to suggest, based on our data, that sleep experts at least, if not GPs too, would like to see *more medicalisation* of sleeplessness as insomnia, if by that we mean better advocacy, ‘ownership’ and medical education, without this necessarily translating into more pharmaceuticalisation, given other non‐pharmaceutical alternatives. Second, returning to the above point about the conceptual ambiguity and fuzziness of ‘insomnia’, it is also tempting to suggest that sleeplessness in general and insomnia in particular, are not simply liminal matters but boundary objects too. Boundary objects that is to say, in Star and Griesemer's (1989) terms, which are both ‘plastic enough’ to ‘travel’ across different boundaries, sites and settings, and ‘robust enough’ to maintain some sort of coherence in so doing, albeit ‘without consensus’. Despite the ‘standardizations’ of biomedicine moreover courtesy of diagnostic systems such as ICD and DSM, ‘insomnia’ still displays a degree of ‘interpretive flexibility’ within and across these different sites or settings.

What further light though, if any, can pharmaceuticalisation shed on these matters? Our data show how the medicalisation and pharmaceuticalisation of sleeplessness intertwine in complex ways, albeit in ways, contra Busfield (2017a,b), which should not be collapsed or conflated one into the other. The conceptual utility of pharmaceuticalisation, in this particular case, is that it directs our attention to important dimensions and dynamics occurring *within* processes of medicalisation. Pharmaceuticalisation in this respect affords us greater conceptual precision and purchase regarding these important dimensions and dynamics that may be obscured through recourse to the concept of medicalisation alone.

Perhaps the key issue here, in this regard, is that taking prescription rates for hypnotics as a proxy for the medicalisation, or pharmaceuticalisation even, of sleeplessness as insomnia is far too simplistic if not problematic. This is because *other pharmaceuticals* with sedating effects are also used to treat sleeplessness when insomnia is viewed as a *symptom of another disorder* (e.g. antidepressants); cases of secondary insomnia, in medical parlance, whether formally diagnosed as such or not, or when over‐the‐counter medicines (e.g. antihistamines) are purchased and consumed.

Few patients in fact, as we have seen, were given a diagnosis of (primary) insomnia through interaction with their GP, which in turn contributes to the incomplete medicalisation of sleeplessness as insomnia conceptually, with implications for the ways in which it is medically managed. As for OTCs, whether or not these can be regarded as instances of the pharmaceuticalisation of sleeplessness *beyond* medicalisation is of course debatable and in need of further investigation to more fully unravel. Either way, the fact remains that just looking a prescription hypnotic rates provides only a partial picture of the pharmaceuticalisation of sleeplessness.

Further important dimensions and dynamics of these processes, particularly their *temporal dynamics*, also come into view in relation to the next question regarding the demedicalisation/depharmaceuticalisation of sleeplessness. Whilst depharmaceuticalisation has yet to occur to any significant degree, despite the aforementioned further decline in prescription hypnotics over the past three years, downward regulatory pressures and countervailing trends are nevertheless increasingly pointing in this direction. Behavioural and psychological measures as recommended first‐line interventions support this assessment.

We have also seen that GPs in our study typically stated that they were *reluctant* to prescribe hypnotics and talked in *moralised* terms as ‘low prescribers’ who saw these medicines as a last resort and as a short‐term measure. This again is unsurprising given studies from the 1980s onwards that have reported GPs’ ambivalence about the prescribing of these pills and willingness to suggest alternatives (Davy *et al*. 2015, Gabe and Lipshitz‐Phillips 1984, Sirdifield *et al*. 2013). The GPs we interviewed certainly recognised the partial and problematic medicalisation of insomnia, especially in relation to lack of resources, secondary care treatment pathways and alternatives to pharmaceuticals. In some respects, therefore, they can be seen as positioning themselves as engaging in *‘reluctant pharmaceuticalisation’* when they prescribed sleeping pills for *chronic insomnia*; a term we would distinguish from Moloney's (2017) notion of ‘reluctant medicalisation’ *precisely because it is reluctance regarding pharmaceuticals rather than medicalisation more generally*.

However, this was not necessarily the case, returning to the previous points concerning insomnia as symptom or disorder, when they prescribed pharmaceuticals to treat insomnia as symptom of another disorder where a short course of hypnotics was viewed as being appropriately prescribed, sometimes alongside other medication, whilst waiting for that to take effect as treatment for secondary insomnia. Moreover, as one of our sleep experts suggests, insomnia persisting beyond the treatment of other disorders (e.g. depression) was one argument put forward for conceptualising insomnia as a discrete or co‐morbid disorder warranting treatment in its own right, rather than as a symptom of something else. *The temporal dynamics of insomnia and medication* therefore came into play here to some extent, with *short courses of 2–4 weeks* seen as legitimate and falling within current guidelines.

In the longer‐term, however, sleep problems associated with these other disorders were treated with pharmaceuticals *other than hypnotics* with sedating side effects. This practice was considered routine by some of the GPs we spoke to, while others were more critical, viewing it as nothing more than a way to reduce the volume of hypnotic prescribing in the practice (e.g. ‘script switching’ where one pharmaceutical is replaced by another).

*Chronic use of hypnotics* was more problematized and moralised and viewed by GPs as the ‘wrong’ type of use, as having little benefit for the patient and as stigmatised in terms of being linked to potential dependency or addiction. It was this group of chronic users who were the target of *depharmaceuticalisation* efforts by GPs, and possibly where sleeplessness/insomnia was subject to a degree of *demedicalisation too*. Chronic insomnia, we found, tended to be positioned as more of a psychological problem with behavioural therapies put forward as treatment options. The extent to which these measures actually amount to depharmaceuticalistion of insomnia to date is questionable though, given the nature of these very problems and the limited alternatives available at present. Here, our respondents cast a critical light on current regulatory policy, service provision and prescribing practice as insomnia patients, particularly chronic patients, may find themselves in a difficult if not *liminal position*, due to the exhaustion of the ‘short term’ treatment options via primary care and lack of access to specialised sleep centres or psychological services, which are scare in the UK (see Gabe *et al*. 2016). Again, there are obvious policy implications to our findings here regarding this group of chronic insomnia patients.

What we have here nevertheless, sociologically speaking, are *countervailing* pressures to depharmaceuticalise, if not demedicalise, insomnia. These in turn are perhaps best conceptualised through related processes of *healthicisation* and *psychologisation*, each of which again has complex and contingent relations with medicalisation^5^. Healthicisation in this case pertains to interventions to improve sleep hygiene and encourage healthier sleep‐wise or sleep‐smart lifestyles. Psychologisation, in contrast, relates to CBTi and other psychological interventions, some of which explicitly promote themselves as non‐pharmaceutical alternatives. Whilst healthicisation then extends beyond medicalisation, psychologisation may or may not do so, depending on the particular mode of psychological intervention and its relationship to medicine.

All in all, to conclude, sleeplessness it seems is only partially and problematic medicalised as insomnia to‐date. Much of this medicalisation moreover, on closer inspection, turns out to be better captured as pharmaceuticalisation, reluctant or otherwise, including the pharmaceuticalisation of sleeplessness beyond prescription hypnotics on the one hand, and countervailing moves in the opposite direction on the other hand, which if not quite amounting to the depharmaceuticalisation of sleeplessness as yet, are nevertheless pointing in this direction in terms of non‐pharmaceutical alternatives. Pharmaceuticalisation therefore, contra recent critics (such as Busfield 2017a,b), has distinct analytical value in directing our attention, in this particular case, to important dynamics occurring *within* if not *beyond* the medicalisation of sleeplessness as insomnia, including potential countervailing moves of a depharmaceuticalising kind in future.

## Notes

1 The PCRN is an organisation within the NHS that works with researchers to promote the successful delivery of research studies in the NHS. As a part of our ethical approval we were given a contact within the PCRN who liaised with NHS primary care staff and patients, inviting them to take part in the research on our behalf. The research team therefore did not have to gain access to staff or patient data or medical records as this was done through a third party. We were allowed access to two Primary Care Trusts (PCTs) as they were then known.

2 National Institute for Health and Care Excellence (NICE) is a UK regulatory body providing national guidance around clinical treatments protocols.

3 Continuous positive airway pressure (CPAP) is a treatment that uses mild air pressure to treat sleep‐related breathing disorders including sleep apnoea.

4 The Improving Access to Psychological Therapies (IAPT) programme began in 2008.

5 See for example Hislop and Arber (2003), Williams (2005), Cheung *et al*. (2017) for previous discussion of these concepts and relations, and Correia (2017) for a recent rethink of medicalisations in the plural.
